# Ultrasound Mediated Microbubbles Destruction Augmented Sonolysis: An In Vitro and In Vivo Study

**DOI:** 10.1155/2017/7021929

**Published:** 2017-08-16

**Authors:** Hai Cui, Qiong Zhu, Yunhua Gao, Hongmei Xia, Kaibin Tan, Ying He, Zheng Liu, Yali Xu

**Affiliations:** Department of Ultrasound, Xinqiao Hospital, The Third Military Medical University, 183 Xinqiao Street, Chongqing 400037, China

## Abstract

**Objective:**

This study was aimed at exploring ultrasound mediated microbubbles destruction (UMMD) assisted sonolysis in both the in vitro and in vivo clots.

**Methods:**

Therapeutic ultrasound (TUS) and lipid microbubbles (MBs) were used in whole blood clots and divided into the control, TUS group, and TUS + MB group. Thrombolytic rates and microscopy were performed. Color Doppler flow imaging (CDFI) and angiography were performed to evaluate the recanalization rates and flow scores in femoral arterial thrombus (FAT) in rabbits. FAT were dyed with H&E.

**Results:**

The average thrombolytic ratios of TUS + MB group were significantly higher than those of TUS group and the control group (both *P* < 0.05). Clots had different pathological changes. Recanalization rates and flow scores in TUS + MB group were significantly higher than the control and TUS group. Flow scores and recanalization ratios were grade 0 in 0% of the control group, grade I in 25% of TUS group, and grade II or higher in 87.5% of TUS + MB group after 30 min sonolysis.

**Conclusions:**

Both the in vitro and in vivo sonolysis can be significantly augmented by the introduction of MBs without thrombolytic agents, which might be induced by the enhanced cavitation via UMMD.

## 1. Introduction

Thrombus formation has become a leading risk factor on the embolization of cardiocerebral organs. It is critically important to perform effective thrombolysis and improve recanalization rate to decrease the rate of mortality and disability and to improve the life quality of the patients [1, 2]. However, the bleeding risk and the expenses of fibrinolytic drugs such as t-PA have limited the wide application of these agents, which hampered the effective rescue of some emergency patients. It is vital and imperative to find a way to solve the problems. Sonolysis (clots lysis using ultrasound and microbubbles) is an approach that induces microbubbles (MBs) oscillations to cause clot disruption and restore perfusion, which could potentially accelerate clots dissolution even without the introduction of drugs.

Therapeutic ultrasound (TUS) could accelerate thrombolysis and recanalize vessel possibly by mechanical subdivision like acoustic cavitation. MBs might enhance ultrasound cavitation and potentially augment sonothrombolysis [3, 4]. Although there were many studies about sonothrombolysis in vitro or in vivo [3–8], the sonolytic treatment using the same TUS apparatus without fibrinolytic drugs which involved both the in vitro and in vivo studies was rare, and the pathological changes of clots and the potential mechanism had not been deeply investigated.

The objective of this study was to determine whether combining TUS with MBs could accelerate thrombolysis at the in vitro and in vivo studies without the use of fibrinolytic drugs. Self-made perfluorocarbon filled lipid encapsulated MBs and TUS at frequency of 1 MHz were applied in both the in vitro clots and FAT in rabbits; the thrombolytic efficacy, pathological changes, and potential mechanism were explored.

## 2. Materials and Methods

### 2.1. In Vitro Study

#### 2.1.1. Preparation of Clots

A total of 500 ml blood was extracted via the elbow vein from 5 healthy people and approved by IRB in our institute. Each blood sample (1 ml) was put to a 1.5 ml centrifuge tube and immersed into a 37° water bath for 5 h, then washed with saline for three times, dried on the filter paper for 10 min, and weighed with electronic balance. Samples with a mass of 200~300 mg were chosen as the thrombolytic objectives.

#### 2.1.2. MBs Preparation

Lipid coated MBs was prepared by the lyophilization of a suspension of two lipids, 1,2-dipalmitoyl-sn-glycero-3-phosphoglycerol (DPPG) and 1,2-distearoyl-sn-glycero-3-phosphoethanolamine (DSPE), and agitated with perfluoropropane gas by a mechanical amalgamator. The mean particle diameter of the MBs was 2 *μ*m, and concentration was (6~9) × 10^9^/mL [9].

#### 2.1.3. TUS Apparatus and Parameters' Settings

TS-A was purchased from Angel Scientifc Ctd, Wuhan, China. The paramaters' settings were as follows: frequency, 1.0 MHz; acoustic power, 0~2.2 W/cm^2^; detector circular area, 10 cm^2^; irradiation mode; and continuous and pulse mode.

#### 2.1.4. Experimental Procedures

Sonolysis treatment was performed in 3 groups: the control group, TUS group, and TUS + MB group (*n* = 20).

#### 2.1.5. Thrombolytic Treatment

**Figure 1:**
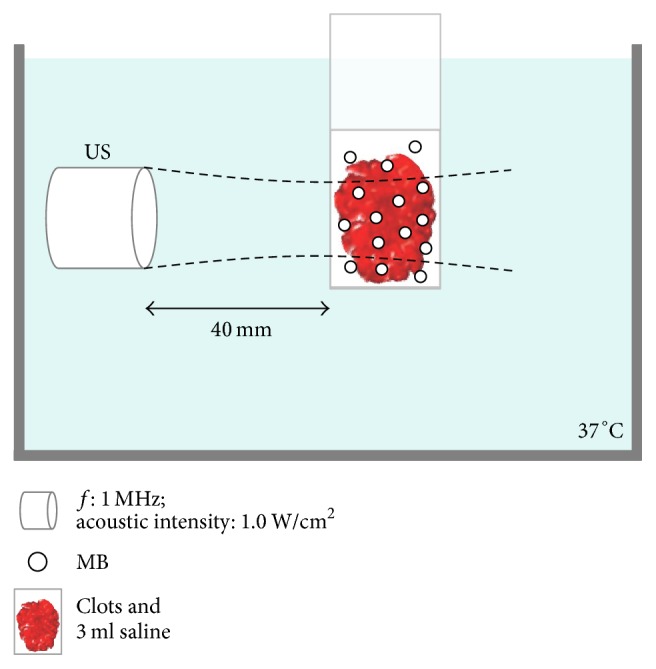
Diagram of experiment setup at in vitro study.

The thrombus aged 5 h was prepared from the fresh blood of 5 donors. The original mass of the thrombus was weighed (*W*_0_) and put into a polyethylene centrifuge tube with a wall thickness about 2 mm. The clots and 3 ml saline were put in the centrifuge tube and incubated in water bath at 37° for 5 h. Ultrasonic parameters were set as follows: frequency, 1 MHz; acoustic intensity, 1.0 W/cm^2^; continuous irradiation for 10 min. Ultrasound probe was located 4 cm far from the thrombus within the degassed water bath (Figure 1). After thrombolysis, all the samples were washed with saline for three times, put on the filter paper to dry for 10 min, and then weighed to get the mass after treatment (*W*_1_). The thrombolytic rate was calculated with the formulae: [(*W*_0_ − *W*_1_)/*W*_0_]  × 100%. The pathological changes of clots following thrombolytic therapy were observed with light and scanning electron microscope.

#### 2.1.6. DiO (3′3Dioctadecyloxacarbocyanine) MBs Preparation and the Distribution in Clots

DiO stained MBs solution preparation: DiO (1 mg) was dissolved in 10 ml DMSO to prepare the DiO solution. DiO solution (10 *μ*l) was mixed with the raw materials of MBs like DPPG and DSPE, fully hydrated and shaken; perfluorocarbon was added and oscillated with mechanical oscillation device, finally washed, and purified. The free DiO was fully washed away with saline and kept standing for 30 min; then the subnatant part of DiO MBs solution was taken out, repeated for 3 three times. The DiO stained MBs were diluted by 50 times, observed under fluorescent microscopy, and used as tracers to detect the MBs in MB_DiO_ group and TUS + MB_DiO_ group (*n* = 10). After TUS irradiated MBs treatment, the samples were cut into 8 *μ*m icy slices. The distributions of MB_DiO_ at the surface and inner part of thrombus were observed under fluorescent microscopy.

#### 2.1.7. Pathological Changes of Thrombi after Treatment under Light Microscopy and Scanning Electronic Microscope

The clot samples of the three groups after treatment were dyed with H&E and observed under light and scanning electron microscope. The surface and inner structure of thrombi and the structure integrity of the fibers were observed and compared among groups.

### 2.2. In Vivo Thrombolytic Experiments

#### 2.2.1. Establishment of FAT Models in Rabbits and Thrombolytic Experiments

A completely obstructed FAT model was made by occluding the right femoral artery (FA) trunk for 2 h with two artery clamps (the first clamp was put at a distal part of FA) in a distance about 1 cm in New Zealand rabbits. Unilateral and complete occlusions of iliofemoral arterial were made in 36 New Zealand rabbits and were grouped into the control group, TUS group, and TUS + MB group (*n* = 12). TUS (1 MHz, 1.5 W/cm^2^, 30 min, continuous irradiation) was performed at the site of the right FAT. Self-made lipid coated MBs (0.05 ml/Kg) were injected via ear vein, followed by the infusion of 3 ml saline.

#### 2.2.2. Assessment of Thrombolytic Efficacy

CDFI (color Doppler flow imaging) and angiography of FAT were performed at 0 min, 15 min, and 30 min during UMMD irradiation to evaluate the recanalization rates and grades in the three thrombolytic groups. The blood flow grading criteria were demonstrated in Table 1.

Recanalization rate: the reperfused FAT graded I or higher assessed by the blood flow grading was recanalized arteries, and the recanalization rates were calculated by the ratios of recanalized arteries from the sum of FAT at 0 min, 15 min, and 30 min in each group (Table 2).

#### 2.2.3. Pathological Exam

The pathological changes of FAT were demonstrated with light microscopy after thrombolytic treatment in different groups.

### 2.3. Statistical Analysis

Statistical analyses were performed using SPSS 13.0 package (SPSS, Chicago, IL, USA). Thrombolytic rates were expressed as mean ± standard deviation (SD). Differences among the three groups were determined by analysis of variance (ANOVA). Recanalization rate was determined by analysis of Fisher's method. *P* values < 0.05 were considered statistically significant.

## 3. Results

### 3.1. In Vitro Studies

#### 3.1.1. Sonothrombolytic Efficacy

**Figure 2:**
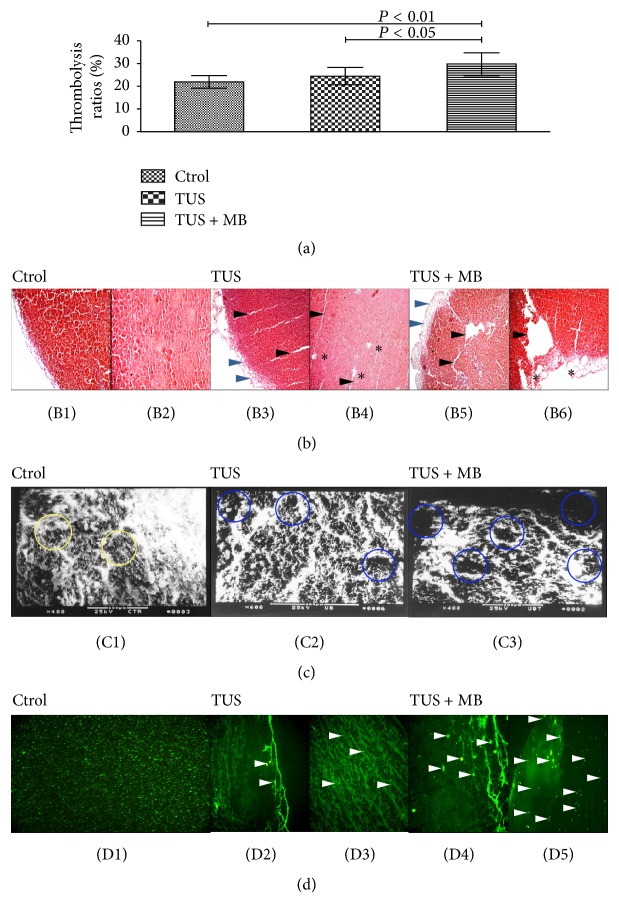
Thrombolytic efficacy and the pathological changes of in vitro clots. (a) Histogram of thrombolytic rate in the three groups in clots. TUS + MB group versus TUS group, *P* < 0.05; TUS + MB group versus the control group, *P* < 0.01. (b) Pathological changes of clots stained with HE under light microscopy. In the control group, the surface and the inner fibrin structure of the red thrombus remained normal without micropores or clefts ((B1)-(B2), ×100); in TUS group, a few tiny fractures and vacuoles were observed at the inner structure (black arrowheads) and loosened surface (blue arrowheads) of clots and a small amount of tiny clefts (black asterisks) was found in an almost intact fiber net within the clots ((B3)-(B4), ×100); in TUS + MB group, there were more and larger vacuoles (black asterisks) at the loosened surface (blue arrowheads) area and more irregular clefts (black arrowheads) and tunnel-like disruption within the clots ((B5)-(B6), ×100). (c) Pathological changes of clots under scanning microscopy. Intact structure and fibrin networks (yellow circles) were observed at the surface and inner part of clots in the control group (C1); there were micropores at the surface, and a loose fibrin network with microcavities (blue circles) at the inner part of clots in TUS group (C2) and even larger cavities (blue circles) and disrupted fiber in TUS + MB group (C3). (d) Fluorescent distribution of MBs in clots following TUS irradiation. DiO stained MBs were in bright green particles and evenly distributed under fluorescent microscopy ((D1), ×200). A bright fluorescent band at the surface of clots ((D2), ×100) and a few DiO-positive MBs (white arrowheads) were observed within the clots ((D3), ×100) in MB group; a brighter and broader band at the surface of clots ((D4), ×100) and more DiO MBs in spherical or irregular particles (white arrowheads) at the surface and inner part of clots ((D5), ×100) in TUS + MB group.

Significant differences were demonstrated in thrombolytic rates between TUS + MB group and the other two groups; a much higher thrombolytic rate was indicated in TUS + MB group (29.51 ± 5.17)% than TUS group (24.14 ± 3.93%, *P* < 0.05) and the control group (21.74 ± 2.78%, *P* < 0.01), Figure 2(a).

#### 3.1.2. Pathological Changes under Light Microscope

Different changes in clots were demonstrated with light microscopy after sonolysis. In the control group, the inner fibrin structure of the red thrombus remained normal without micropores or clefts; a small amount of tiny clefts was found in an almost intact fiber net within the clots, and a few tiny fractures and vacuoles were observed at the loosened surface of clots in TUS group; there were irregular clefts and tunnel-look destruction within the clots and more and larger vacuoles at the surface area in TUS + MB group (Figure 2(b)).

#### 3.1.3. Pathological Changes under Scanning Electronic Microscope

Intact structure was observed at the surface of clots in the control group; there were micropores at the surface and a loose fibrin network with microcavities at the inner part of clots in TUS group and even larger cavities and disrupted fiber net in TUS + MB group (Figure 2(c)).

#### 3.1.4. Pathological Changes under Fluorescent Microscope

DiO stained MBs were in bright green particles and evenly distributed under fluorescent microscopy. There was a bright fluorescent band at the surface of clots and a few DiO-positive MBs within the clots in MB group; a brighter and broader band at the surface of clots and more DiO MBs in spherical or irregular particles were presented in TUS + MB group (Figure 2(d)).

### 3.2. In Vivo Study

Total obstructive clots were built in the right femoral arteries in 36 rabbits by color Doppler flow imaging (CDFI). Recanalization rate and scores were assessed with CDFI and femoral artery angiography.

#### 3.2.1. Thrombolytic Efficacy Assessed with Revascularization Rate with CDFI and Angiography

Revascularization rate was evaluated with CDFI and angiography; there were no revascularization in FAT in the control group at 0 min, 15 min, and 30 min; there were 25% recanalization at 30 min in TUS group and no reperfusion at 0 min and 15 min; 25% reperfusion at 15 min and 87.5% reperfusion at 30 min in TUS + MB group; significant difference was noted between TUS and TUS + MB group, *P* < 0.05 (Table 1).

#### 3.2.2. Flow Score Assessment with CDFI

**Figure 3:**
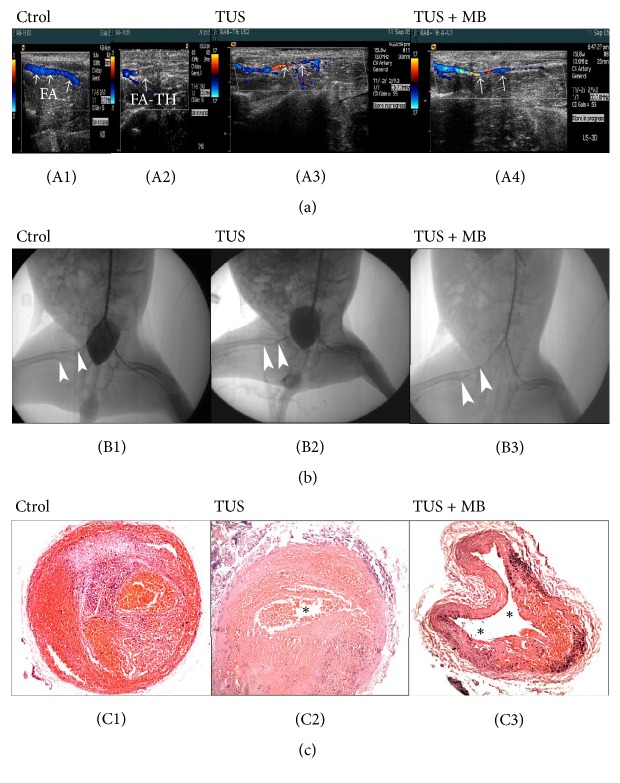
Assessment of thrombolytic efficacy and the pathological changes in FAT in rabbits. (a) Assessment of thrombolytic rate with CDFI. In the control group, continuous flow was recorded in the left femoral artery ((A1), white arrows), while a defect in blood flow was found at FAT in the right femoral artery with CDFI ((A2), white arrows); a narrow and strip flow in blue or turbulent in grade I was observed through the edge of clots in TUS group at 30 min ((A3), white arrows); blood flow of grade II or higher ((A4), white arrows) was recorded at 30 min in the TUS + MB group. (b) Assessment of thrombolytic rate with X-ray angiography. No reperfusion in the control ((B1), white arrowheads), grade I flow in TUS group ((B2), white arrowheads) and grade II ((B3), white arrowheads) in TUS + MB group at 30 min. (c) Pathological changes of FAT following sonothrombolytic treatment under light microscopy. An obstructive thrombus consisted of red blood cells and fibrin matrix was observed (C1); small amount of red blood cells destruction was observed associated with partially dissolved FAT ((C2), black asterisk) at 30 min in TUS group; clots were sonolysed in large amount and the recanalized FA was collapsed with residual thrombus in the inner wall of FA ((C3), black asterisks).

In the control group, a defect in blood flow with CDFI was found at FAT in the right FA at 0 min, 15 min, and 30 min following thrombolytic treatment; a narrow and strip flow in blue or turbulent in grade I was observed through the edge of clots in 25% reperfused rabbits in TUS group at 30 min; blood flow of grade I in 25% at 15 min and grade II or higher in 87.5% reperfused FAT was recorded in TUS + MB group at 30 min following sonolysis treatment (Figure 3(a)).

#### 3.2.3. Assessment with X-Ray Angiography

Angiography was performed in FAT in rabbits following CDFI. The reperfusion rates and the flow scores were assessed at 30 min of therapy. A similar result was demonstrated as follows: no reperfusion in the control, 25% reperfusion in grade I in TUS group, and 87.5% grade II in TUS + MB group at 30 min (Figure 3(b)).

#### 3.2.4. Pathological Changes of FAT following UMMD Treatment

Under light microscopy, an obstructive thrombus consisted of red blood cells and fibrin net was observed in the control group; FAT were partially dissolved associated with small amount of red blood cells destruction in TUS group; recanalization was demonstrated in FAT in TUS + MB group, large amount of FAT was sonolysed, and the recanalized FA was collapsed with residual thrombus at the inner wall of FA (Figure 3(c)).

## 4. Discussion

Early and effective reperfusion is the key for prompt ischemic tissue rescue and further good clinical outcomes. Ultrasound has been tried on the augmentation of thrombolysis at both in vivo and in vitro studies [10, 11]. Various ultrasound energy levels (spatial peak temporal average intensities of 0.2~2.0 W/cm^2^) and frequencies (20 kHz~2 MHz; unfocused transducers) have been used in the former studies [6–8, 10, 11]. The present study aimed to try 1 MHz TUS device and self-made lipid coated MBs in both the in vitro and animal studies to detect the efficacy of UMMD on sonolysis, without fibrinolytic drugs.

### 4.1. Differences in Intensities and Irradiation Time in In Vitro and In Vivo Studies

Frequency, intensity, and irradiation time are important parameters on the biological effect of TUS. The same frequency, different intensities (in vitro, 1 W/cm^2^; in vivo, 1.5 W/cm^2^) and various irradiation durations (in vitro, 10 min; in vivo, 30 min) were tried in the studies. In in vitro study, red thrombus aged 5 h and MBs were put in a static system and irradiated with a 1 MHz frequency TUS and 4 cm irradiation distance, a relatively lower intensity of 1 W/cm^2^, and lesser exposure time of 10 min could make a good fibrinolytic effect, while a higher power of 1.5 W/cm^2^ and longer irradiation time of 30 min in 2 h obstructive FAT in rabbits also got a good performance in recanalized rates and scores, without the use of fibrinolytic agents. The intensity was higher and exposure time was longer in FAT than those in clots, since a completely blocked clot aged 2 h might need higher power to dissolve; and the intravenous injected MBs may be obstructed by the circulating organs; thus the MBs enhanced cavitation was not so remarkable as the in vitro stated system. Based on the results in FAT rabbits, we can tell that a longer irradiation for 30 min may result in an improved revascularization rate and grades than 15 min exposure duration in both the TUS + MB and TUS groups. Besides the intensity and frequency, attention should be paid to irradiation time of TUS as well.

### 4.2. Mechanism of UMMD Assisted Thrombolysis

Mechanisms of sonolysis remained unclear [12]. Acoustic cavitation is generally acknowledged as playing a significant role in ultrasound-accelerated fibrinolysis. Cavitation could be produced close to the clot under ultrasound exposure, and the asymmetric collapse of inertial cavitation bubble can lead to intense localized stress forces and the production of a high-speed microjet toward the surface of the clot to disrupt fibrin networks and resulted in thrombus disintegration [13]. Introduction of MBs can lower the ultrasound energy threshold needed to induce acoustic cavitation and increase the lytic activity [3]. MBs can penetrate into fibrin clots to form tunnels with a diameter of 9~35 *μ*m according to the acoustic radiation force [14]. The influential factors of sonothrombolysis included the size of MBs and some parameters of ultrasound like frequency, intensity, and PRF [15]. The present in vitro study has demonstrated that sonolysis could be greatly accelerated without fibrinolytic agents. As evidenced by our pathological findings, there were more fluorescent spots within the clots of TUS + MB group than that of MB group, which proved that TUS could drive or burst much more MBs into the internal slits of clots and produced internal blowing up. Much more micropores, clefts, or cavities induced by sheer forces and microstreaming of TUS were presented at the inner part of clots and larger vacuoles caused by cavitation were observed at the surface area of thrombus under light microscope, indicating both the mechanical effect (sheer forces and microstreaming) and cavitation played an important role on clots sonolysis.

In animal study, a completed occlusive FAT was successfully made in rabbits, which is similar to the situation of arterial thrombus in clinical practice and more applicable for the patients. The reperfusion of blood flow was performed with ultrasound and angiography, and the clot disruption was demonstrated with pathological changes. Higher flow grades and revascularization rates were found in TUS + MB group than TUS group, with an extension of irradiation time; an even greater thrombolytic efficacy was obtained following TUS irradiation with or without MBs. In TUS plus MB group, a flow grade II or higher was demonstrated in 87.5% FAT in TUS + MB group at 30 min, while only 25% reperfusion rates in grade I in TUS group at 30 min and in TUS + MB group at 15 min were presented. These results indicated that MBs could greatly improve the reperfusion rates and scores, and the sonolytic efficacy became even more remarkable with the prolongation of exposure time. Partial clots dissolution was observed under light microscopy in both the TUS and TUS + MB groups, especially in TUS + MB group, which confirmed the efficacy of sonolysis. It is good news for those patients suffering from thrombosis who are at risk of bleeding where large amount of thrombolytic drugs is not applicable and affordable. Researchers [16] have got a similar result that successful microbubble sonolysis without tissue-type plasminogen activator was repeated in a rabbit model of acute ischemic stroke. Brown et al. [17] have got a similar conclusion with an effective loss of clot assessed with reduced infarct volumes and intracerebral hemorrhage at very low dose or even no dose tPA when combining tPA and MB in acute strokes in rabbits.

Both the in vitro and in vivo studies have found out that sonolysis efficacies were greatly improved following UMMD without thrombolytic agents. Since no comparisons of sonolysis associated with intravenous thrombolytic agents and sonolysis with microbubbles were made in the present study, the comparative efficacy of these two methods needs further study.

Based on our results, UMMD could greatly reduce the dosage of fibrinolytic agents; thus the cost and the risk of bleeding of patients were decreased, which is appropriate for patients in poor coagulation function and who are ineligible for fibrinolytic agents. In the future, we plan to emphasize the thrombolytic effect and its risk of bleeding at different dosages of fibrinolytic drugs in some big animal studies.

## 5. Limitations

Different TUS exposure duration and intensities were not tried on in vitro study. Thrombolytic agents of different dosages were not introduced in animal study and should be studied in the future.

## 6. Conclusions

TUS at 1 MHz frequency mediated lipid MBs can augment sonolysis without thrombolytic agents in both the in vitro and in vivo clots. It may be a valuable adjunct to reperfusion therapy for patients ineligible for fibrinolytic agents.

## Figures and Tables

**Table 1:** Flow scores on revascularization in FAT rabbits.

Flow scores	Ratios of revascularization size with FAT
0	No revascularization
I	Small strip flow revascularization
II	Grade I ≤ revascularization flow < 1/3 FAT
III	1/3 FAT ≤ revascularization flow < 2/3 FAT
IV	Revascularization flow ≥ 2/3 FAT

**Table 2:** Revascularization grades and rates with CDFI at different time points (grades (rates)).

Time point (min)	The control group	TUS group	TUS + MB group
0	0/0	0/0	0/0
15	0/0	0/0	I (3/12)
30	0/0	I (3/12)	≥II (9/12)
